# Modeling Quantum Kinetics in Ion Traps: State‐changing Collisions for OH^+^(${}^{3}\Sigma^{-}$ ) Ions with He as a Buffer Gas

**DOI:** 10.1002/cphc.201800119

**Published:** 2018-06-21

**Authors:** L. González‐Sánchez, R. Wester, F.A. Gianturco

**Affiliations:** ^1^ Departamento de Química Física University of Salamanca Plaza de los Caídos sn 37008 Salamanca Spain; ^2^ Institut für Ionenphysik und Angewandte Physik Universität Innsbruck Technikerstr. 25 A-6020 Innsbruck Austria

**Keywords:** inelastic collisions, intermolecular potentials, collisionally inelatic rates, rotational relaxation times, molecular dynamics in cold traps

## Abstract

We present quantum scattering calculations for rotational state‐changing cross sections and rates, up to about 50 K of ion translational temperatures, for the OH^+^ molecular ion in collision with He atoms as the buffer gas in the trap. The results are obtained both by using the correct spin‐rotation coupling of angular momenta and also within a recoupling scheme that treats the molecular target as a pseudo‐(${}^{1}\Sigma^{+}$ ) state, and then compares our findings with similar data for the OH^−^(${}^{1}\Sigma^{+}$ ) molecular partner under the same conditions. This comparison intends to link the cation behaviour to the one found earlier for the molecular anion. The full calculations including the spin‐rotation angular momenta coupling effects have been recently reported (L. González‐Sánchez and R. Wester and F.A. Gianturco, Mol.Phys.2018, DOI 10.1080/00268976.2018.1442597^1^) with the aim of extracting specific propensity rules controlling the size of the cross sections. The present study is instead directed to modelling trap cooling dynamics by further obtaining the solutions of the corresponding kinetics equations under different trap schemes so that, using the presently computed rates can allow us to indicate specific optimal conditions for the experimental setup of the collisional rotational cooling in an ion trap for the system under study.

## 1 Introduction

The trapping and cooling of atomic and molecular particles, which started more than thirty years ago^2^,^3^ has completely transformed the experimental machinery available to atomic and molecular physics studies. The breadth of its scientific impact is really outstanding, ranging from the creation of new quantum systems, observation of novel collisional processes, enhancements in precision measurement techniques and new approaches to quantum simulation and quantum information. One of the most successful techniques involves buffer‐gas cooling,^4^ and references quoted therein, which relies on collisions with cold buffer gas atoms to thermalize a variety of partners like atoms and molecules, neutral and/or ionic, to low temperatures within a confined environment. The buffer gas serves to dissipate translational energy of the target species and, in the caseof molecules, their rotational energy as well. Since this dissipation scheme does not depend on any particular energy pattern, any target species is in principle amenable to it: hence the general applications of this method to a wide variety of molecular systems.^5^ We could therefore reasonably expect buffer‐gas cooling to successfully thermalize the target's rotor to the buffer's low temperature already achieved in the trap.

A central issue with the technique of buffer gas cooling is, in fact, the varying efficiency of cooling different internal degrees of freedom of the molecular target species (e. g. rotations, vibrations, spin‐flips, etc) since the different statistics obviously can have drastic effects on the operational populations of the molecules in a desired state. It is therefore often convenient to use the buffer gas to cool only specific degrees of freedom, leaving others out of thermal equilibrium.

Generally speaking, collision‐induced quenching is more efficient for rotational than vibrational internal degrees of freedom. The former is driven by the orientational anisotropy of the helium interaction with the molecular species: the vibrational motion is therefore typically much faster than a cold collision with the helium buffer gas.^4^ Thus, one can say that typical inelastic cross sections by cold (∼1 K) He atoms could yield rotational quenching every 10–100 collisions, whereas it would take around 10^8^ or inelastic collisions before a vibrational quenching step takes place.^6^ One can therefore reach thermalization for the rotational temperature while leaving the vibrational temperature out of equilibrium. Furthermore, since rotational and translational energy transfer cross sections are similar, thermalization of both degrees of freedom happens fairly rapidly and in tandem during buffer gas cooling experiments.

In a recent study carried out in our laboratory, for example, specific rotational state‐changing collisions at low temperatures have been analysed forhydroxyl anions with He as a buffer gas during photodetachment experiments that manipulated molecular quantum states by non‐resonant processes.^7^ Rather good agreement was found there with the quantum modeling of the relevant dynamics and with our quantitative estimate of the involved collisional rates. The same molecular anions OH^−^/OD^−^, were further computationally modeled under similar conditions in a cold ion trap containing sympathetically cooled Rb atoms,^8^ thereby providing further information on the size and efficiency of the molecular cooling processes for its rotational levels under different environments.

Another system of great current interest for state‐selective experiments in traps is the corresponding cation of the same hydroxyl molecule: the OH^+^(${}^{3}\Sigma^{-}$ ), a positive ion of marked importance for the modeling, among other things, of the chemical abundances of light hydrides in the interstellar medium (ISM), where they are considered to be the first steps of the chemical cycles producing, among other species, the H_2_O molecule.^9^ The rotational line emission of OH^+^, in fact, has been detected toward the Orion bar,^10^ planetary nebulae hosting hot central stars^11^ and toward the Supernova remnant of the Crab Nebula.^12^ The non‐detection of H_2_O^+^ and H_3_O^+^ line emissions in these environments indicates that OH^+^ lines arise in gas layers where most hydrogen is in atomic form, and therefore the hydroxyl cation should be formed by destruction reactions of H_2_ with O^+^: O^+^+H_2_→OH^+^+H.

A detailed study of such reaction has been carried out recently in a comprehensive computational analysis of its rates with their applications tovarious astronomical sources,^13^ indicating the importance of forming OH^+^ in excited rovibrational states. This work also carried out the evaluation of a new potential energy surface (PES) involving OH^+^(${}^{3}\Sigma^{-}$ ) and He atoms, together with a selection of inelastic collision rates for that system.

In a much earlier study on the specific behaviour of OH^+^(${}^{3}\Sigma^{-}$ ) in ultracold ion traps involving He as a buffer gas,^14^ we had analyzed the interaction potential of these species by carrying out an extensive ab initio analysis and further illustrated the possible existence of propensity rules among the state‐changing collisional cross sections involving either rotational or spin‐flipping transitions within the partners. We analyzed the ensuing rates only within the domain of the ultracold regimes of the *μ*K in the trap,^14^ so that no results were presented there at the actual regimes of the ion traps of interest in the present study.

The possibility of investigating the collisionally inelastic behaviour of the OH^+^ cation in cold traps, at temperatures around a few kelvins and under the interaction with He atoms as collisional state‐changing enablers in the trap, is therefore still an open question, both experimentally and from the point of view of the quantum modelling of the relevant processes. Since the corresponding experiments are currently under preparation in our laboratory in Innsbruck, we intend to provide with the present work detailed quantum dynamics calculations which would be able to answer the following questions:

- what are the relative sizes of the collisionally state‐changing cross sections and rates that involve a few of the lower rotational levels of the present cation for temperatures up to about 50 kelvins?;
- can one reliably employ for this open‐shell system the recoupling angular‐momentum scheme successfully, used by us before with other ionic molecules,^15^,^16^ in order to obtain state‐changing rates involving only the total rotational angular momentum?;
- how does the hydroxyl cation's behaviour compare with our earlier findings^7^ for the corresponding anionic system studied experimentally in our earlier work?;
- can we use the calculated rates to model the overall kinetics of the time‐evolution involving the rotational level populations of the lower few states under the expected trap conditions?

A further topic of interest in studying the inelastic collisional dynamics of the present system is provided by searching for possible propensity rules that would allow us to calibrate the relative sizes of the state‐changing cross sections in the presence of the full couplings of the electronic spin and the rotational angular momenta, within the full angular momentum coupling dynamics. We have recently carried out such a study for this very system and have been able to computationally show the dominance of the pure spin‐changing collisional probabilities^1^ in relation to the other rotationally inelastic channels. In the present analysis, however, we shall extend the use of the quantum dynamics to further discuss the role of its computed rates within the master eq.s that analyse the temporal evolutions of the rotational state populations under a variety of conditions chosen to describe the ion trap and of which those discussed above have been an example.

Hence, the present paper will be organized as follows:

- Section 2 briefly outlines the calculations of the PES for the title system and compares our earlier results with the more recent ones;
- Section 3 provides a short summary of the well‐known time‐independent quantum dynamics equations and their implementation in the present study;
- Section 4 reports the calculated cross sections for both excitations and cooling inelastic collisions between the significant rotational levels of OH^+^ partners;
- Section 5 discusses the rates over the expected range of temperatures of the trap, while Section 6 reports the evaluation of the relaxation times provided by the rates equations at different trap conditions for the He buffer density and the initial trap temperature.

Our present conclusions are finally given in our Section 7.

## 2 The Computed Interaction Potentials

The anisotropic interaction in the $\left[R,\theta \right]$ Jacobi space for the hydroxyl cation with the neutral ^4^He atom was computed in our group a while ago, treating the target molecule as a rigid rotor (i. e. at its fixed equilibrium bond distance of r_*eq*_=1.0279 Å^15^). These calculations were carried out at the post Hartree‐Fock level using the MP4 formalism within a basis set expansion of the aug‐cc‐pVQZ quality which further included the basis‐set‐superposition‐error (BSSE) corrections via the counterpoise method.^16^ The employed set of Jacobi coordinates comprised 12 values of the θ angle between 0° and 180°, and covered a radial range of values from 2.0 to 10.0 Å, for a total grid of 320 radial points. The details of the fitting procedure were given before^14^,^15^ and will not be repeated here. The long‐range (lr) region of radial values was connected to the fitted values of the above short‐range (sr) region by ensuring that the overall interaction includes asymptotically the leading dipole polarizability of the neutral atomic partner plus the dipole‐polarization terms:

(1) $$V_{lr}\left(R,\theta \right)∼\frac{\alpha_{0}}{2R^{4}}+\frac{2\alpha_{0}\mu }{R^{5}}cos\theta$$

where $\alpha_{0}$ =1.41 a${}_{0}^{3}$ is the polarizability of the helium atom and *μ* is the permanent dipole of the OH^+^ partner.

The calculations for a new PES were recently carried out again in a later study,^13^ where the fuller $\left[R,r,\theta \right]$ Jacobi space was considered. The radial distances corresponded to 57 R values between 2.75 a_0_ and 32 a_0_, with a varying step as the earlier calculations. The intramolecular distance r varied between 1.7 and 2.6 a_0_ over a grid of five points, while the angular variable θ was given by a grid of 15 Gauss‐Legendre nodes. The employed basis set expansion was also an aug‐cc‐avQZ within an unrestricted CCCD(T) method as described in^17^ and implemented by the MOLPRO suite of codes.

As is well‐known one can further represent the full PES via the radial multipolar coefficients, which originate from the standard expansions carried out for the two PES for the case of the target's r_*eq*_ geometry, obtained by explicitely writing:

(2) $$V\left(R,\theta |r_{eq}\right)=\sum_{\lambda }^{\lambda_{max}}\;V_{\lambda }\left(R|r_{eq}\right)\;P_{\lambda }\left(cos\theta \right)$$

**Figure 1:**
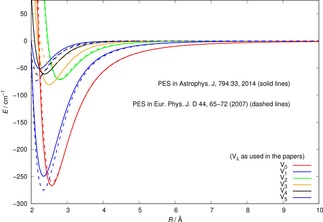
Comparing the computed multipolar coefficients from the existing PESs for OH^+^/He. The solid lines are from ref [13], while the dashed lines are from ref [14].

A comparison between such radial coefficients is given for the lower six values of their *λ* index in Figure 1, while the *λ_max_* values employed to convergence in the above eq. were carried up to 15 in both expansions.

We clearly see from that comparison that both calculations, in spite of being nearly 10 years apart, are producing two PES for the target at its r_*eq*_ which are very similar to each other. The dominating terms are those with *λ*=0,1 and are essentially coincident in the two sets of calculations, while small differences visible between the two calculations around the well regions of their higher anisotropic contributions are only of the order of a few cm^−1^. The detailed features of orientational properties of the interaction are given more extensively in refs [13, 14, 15]. In the present calculations we have finally decided to employ the more recent PES, provided to us by Dr F. Lique.^13^

## 3 Multichannel Quantum Dynamics

The present molecular ion has a ${}^{3}\Sigma^{-}$ electronic configuration, hence its physical rotational levels are split by spin‐rotation coupling dynamics that requires the electronic spin angular momentum to be coupled to the molecular rotational angular momentum. Following the Hund's case (b), we can write that the electronic spin angular momentum **S** couples with the nuclear rotational angular momentum **N** to form the total angular momentum **j** :

(3) j=N+S

It thus follows that each j level (with j≥ 1) is split into three subleves F_1_, F_2_, F_3_ which have the following rotational wavefunctions:

(4) $$\left|F_{1}\;j\;m\right\rangle =\left|N=j-1,S\;j\;m\right\rangle \left|F_{2}\;j\;m\right\rangle =\left|N=j,S\;j\;m\right\rangle \left|F_{3}\;j\;m\right\rangle =\left|N=j+1,S\;j\;m\right\rangle$$

where $\left|m\right\rangle$ defines the projection of **j** along the space‐fixed z‐axis.

For a given value of N, the molecular rotational quantum number, the corresponding relative energies for the three levels defined in eq 5, and associated with the three different rotational wavefunctions, are given by:^18^

(5) $$E_{j=N+1}=BN\left(N+1\right)-DN^{2}{\left(N+1\right)}^{2}-\frac{2\lambda N}{3(2N+3)}+\gamma NE_{j=N}=BN\left(N+1\right)-DN^{2}{\left(N+1\right)}^{2}+\frac{2\lambda }{3}-\gamma E_{j=N-1}=BN\left(N+1\right)-DN^{2}{\left(N+1\right)}^{2}-\frac{2\lambda (N+1)}{3(2N-1)}-\gamma \left(N+1\right)$$

where *B* is the rotational constant, and the other constants describe: *D* is the centrifugal distortion, *λ* the spin‐spin interaction, and *γ* the spin‐rotation. The spin angular momentum and the rotational angular momentum are those already described and discussed for the previous eq.s and clearly involve the structural characteristic of the ionic molecular target.

**Figure 2:**
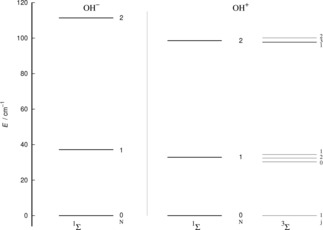
Computed lower‐lying rotational level spacing for the OH^+^ rigid rotor. The column on the right presents the levels splitting for the ${}^{3}\Sigma$ case, while the one on the left shows the pseudo‐${}^{1}\Sigma$ case where $\left|N\right\rangle$ in the only rotational quantum number.The case of the negative anion system is shown on the third column of the extreme left.

The rotational energy levels of the OH^+^ molecule were computed using the experimental constants provided earlier to us by Brown^14^ and the energies of some of the lower fine‐structure levels are given by Figure 2. Due to its relative smallness, the centrifugal distortion has been neglected in the present calculations.

One clearly sees from the relative energy spacings shown by Figure 2 that the ΔN≠ 0 transitions involve much larger energy gaps than those transitions were only the **S** quantum number changes: spin‐flip processes therefore involve much smaller energy transfers during inelastic collisions, as we shall further discuss below and as we have already illustrated in our earlier work.^1^ We also see in that same Figure 2 that, by removing the spin‐rotational coupling which causes the energy splitting terms, we could obtain an approximate picture for the rotational structure of the target molecule that becomes essentially described as a pseudo‐${}^{1}\Sigma$ case, with the corresponding simplification of the energy spacings reported by the schematic energy ladder in the center of Figure 2. The validity of such a recoupling scheme was recently tested by us for the Hydrogen Molecular Ions (HMIs) in collision with He^19^ and found to provide a very realistic description of the inelastic dynamics.

To further treat the quantum dynamics for the target molecule in a specified state $\left|F_{i}jm\right\rangle$ which interacts with the He atom as a structureless partner, we need to generate the scattering eigenfunctions of the total angular momentum **J**, which describe the total collisional system, and which can be written as:^20^

(6) $$\left|F_{i}jLJM\right\rangle =\sum_{MM_{L}}\;\left\langle jmLM_{L}|JM\right\rangle \left|LM_{L}\right\rangle \left|F_{i}jm\right\rangle$$

Here <····|··>|··> is a Clebsch‐Gordan coefficient, *M* is the projection of *J* along the space‐fixed *z* axis, *L* is the relative orbital angular momentum quantum number associated with the impinging spherical helium atom, *M_L_* is its projection along *z*, and $|LM_{L}>$ is the wave function for the angular motion of the helium structureless particle. The index *F_i_* refers to the labelling of the splitted spin levels (*i*=1,2,3) reported in equation 5. The target rotational wave functions of equation 6 may also be expressed in a general form

(7) $$\left|F_{i}jm\right\rangle =\sum_{N=j-1}^{N=j+1}\;c_{NF_{i}}^{j}\left|NSjm\right\rangle$$

To now solve the coupled‐channel (CC) equations we need to generate the transition matrix elements between the fine‐structure states defined by 7 which are now coupled via the anisotropic PES discussed in the previous Section. One should note here that the quantum dynamics involves a potential coupling which is only electrostatic in nature and therefore does not directly involve spin‐flipping effects.^19^,^20^ Such effects are only indirectly brought in by the recoupling of the changed rotational quantum numbers, during a state‐changing collision (ΔN≠ 0), with the electronic spin angular momentum to produce a different set of coefficients of the changed *F_i_* values in eq 7. We shall further discuss this effect later on in this work. For a fixed value of J, the relevant potential‐coupled matrix elements are given as^14^

(8) $$\langle {F{}^{'}}_{i}j{}^{'}L{}^{'}JM\left|V\right.\left|F_{i}jLJM\right\rangle =\sum_{NN{}^{'}}\;c_{N{}^{'}{F{}^{'}}_{i}}^{j{}^{'}}c_{NF_{i}}^{j}\langle N{}^{'}S{}^{'}j{}^{'}L{}^{'}JM\left|V\right.\left|NSjLJM\right\rangle$$

Where the matrix elements of *V* between pure Hund's case (b) basis functions are given by $\langle N{}^{'}S{}^{'}j{}^{'}L{}^{'}JM\left|V\right.\left|NSjLJM\right\rangle$ .^19^,^20^

We have carried out calculations by solving the relevant coupled‐channel equations for all the initial states from *N*=0 to *N*=5 using our in‐house quantum scattering code ASPIN,^21^ and solved both the full fine‐structure coupling scheme of the ${}^{3}\Sigma^{-}$ target and the pseudo‐closed shell (i. e. spin zero) simplified coupling scheme of the ${}^{1}\Sigma$ target.^19^ The total angular momentum values were extended, when needed, up to J_*max*_=200. Several closed channels were included in order to obtain convergence of the collisional cross sections: the range of integration was extended, in some cases, out to a radial distance of 300 Å maximum and at least ten closed channels were included in each expansion.^21^ The final cross sections are expected to be converged within 0.1 % of their values.

## 4 Computed Inelastic Cross Sections

As mentioned earlier, we had carried out cross section calculations for the OH^+^/He system a long time ago,^14^ concentrating on ultra‐low collision energies down to 10^−6^ cm^−1^ and analyzing the relative sizes of spin‐flipping and pure‐rotational inelastic cross sections. More recent calculations on the same system^13^ examined the state‐changing cross sections to higher collision energies up to 1200 cm^−1^. A first evaluation of the numerical quality of our results could therefore be had by comparing them with the more recent calculations which are closer to the range of collision energies of interest in the present work.

**Figure 3:**
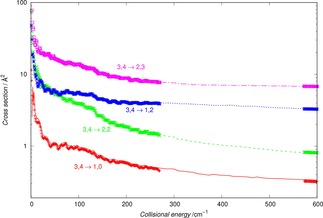
Computed state‐changing cross sections using the fine‐structure angular momentum coupling within the CC dynamical equations. The thin lines report theearlier calculations of ref. [13] while the thick dots show our present calculations. Both sets of cross sections were obtained using the PES described in Section 2.

The results presented in Figure 3 provide such a comparison for a selected set of inelastic cross sections within the full ${}^{3}\Sigma^{-}$ description of the ionic molecular rotor.

We clearly see that the two sets of calculations are essentially coincident and that the same level of numerical convergence, although using different scattering codes, has been achieved for the two sets of computed cross sections.

Another interesting test for the calculations is to verify whether the lack of direct spin‐rotation coupling during the potential‐driven dynamics can generate pseudo‐${}^{1}\Sigma$ cross‐sections (i. e. zero spin cases) which are realistically close to the ${}^{3}\Sigma$ case summed over all its components. It would imply, in fact, that the state‐changing dynamics which would be more likely to be observed in a cold trap, i. e. those involving the larger energy‐transfer values given by changing the rotational quantum number N, can be correctly obtained by disregarding for the moment the reorientation effects that would produce spin‐flipping cross sections, much harder to observe because of the smaller values of their involved energy transfers.

**Figure 4:**
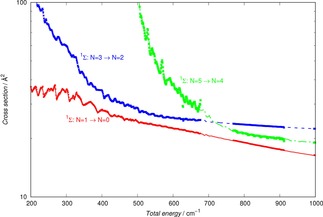
Comparison of computed state changing cross sections within the pseudo‐singlet dynamics, labelling each of the curves, and the summed values obtained with the full fine‐structure coupling dynamics (thick dots and thinner dashes respectively).

The comparison is presented by the results of Figure 4, where the thick dots report the pseudo‐singlet state cross sections, with transition labels next to each of thereported curves. The thin sets of dashes show, over a selected range of energies, the full triplet‐case cross sections summed over their j‐level components.

It is clear from the data reported in that Figure how the recoupling scheme produces rotationally inelastic cross sections with the same energy profiles as those obtained by using the full spin‐rotational angular momentum coupling dynamics. This means that for the present modeling of rotational state‐changing kinetics within the cold traps we could safely focus on using the pseudo‐singlet collisional dynamics approach, expecting that the latter will yield cross sections that are of the correct size in comparison with the calculations which use spin‐rotation coupling coefficients.

**Figure 5:**
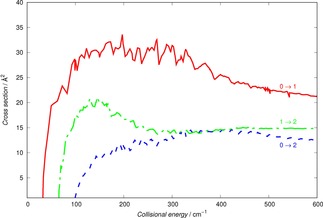
Computed inelastic, rotational state‐changing collisional excitation cross sections between the first three rotational states of the pseudo‐${}^{1}\Sigma$ OH^+^ molecule. See main text for further details.

**Figure 6:**
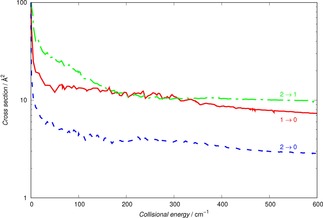
Collisional rotational de‐excitation cross sections computed over the same range of energies as those in Figure 5 and for the same three lowest rotational states of the pseudo‐${}^{1}\Sigma$ ionic molecular target.

Since we expect that the current experiments in our laboratory would follow the pattern already discussed of the OH^−^/He system,^7^ we initially focus on the dynamics between the lowest three levels for N=0,1 and 2 within the pseudo‐closed shell scheme. The results of our computed cross sections are reported by Figure 5 for the excitation processes between the three levels, while the results given by Figure 6 involve the corresponding de‐excitation processes. The collision energies we have considered go from the thresholds for the opening of each of the channels (or from near‐zero collision energies) up to 600 cm^−1^. We expect, as we shall show later, that such range of energies will allow us to obtain the corresponding rates up to about 50 K of temperature in the traps.

All the reported excitation cross sections show a rather marked oscillatory structure originating from collisional resonances, even for collision energies well above the threshold‐opening values. It would be interesting to analyze such structures more in details, but such a task is at the moment outside the scope of the present study, while we expect to possibly carry it out in a following publication.

The data of Figure 5 also show the dominant role of the (0→1) excitation process, while the (1→2) cross sections (also with ΔN=1) are about 50 % smaller over the whole range of examined energies. As expected, the ΔN=2 cross sections are clearly the smallest cross sections, although becoming closer in size to the (1→2) cross sections as the collision energies increase well beyond thresholds. Such a behaviour could be linked to an energy‐gap effect: the (0→1) transition involves an energy transfer value which is more than 50 % smaller that the value for the (1→2) excitation, which is in turn not much smaller than the one for the (0→2) excitation. Thus, as the collision energy increases the last two excitation processes involve transferring energy values that are always larger than the (0→1) process. The latter therefore remains the most favoured excitation process of the three we are considering.

The data of Figure 6 report now the collisional cooling of the rotational levels of OH^+^ over the same range of energies examined by Figure 5. We clearly see the dominance of the ΔN=‐1 processes over the ΔN=‐2 situations. The energy gap plays here a less significant role as for the excitation processes since the (1→0) and (2→1) cross sections only differ from each other at the onset of the processes, while becoming of the same size as collision energy increases.

Because of the similarities between the anion and the cation of the hydroxyl molecule, and since the anion has already been studied in cold traps with He as the buffer gas,^7^ it is interesting to compare the behaviour of their collisional cooling cross sections under the same dynamical conditions and with the same buffer gas.

**Figure 7:**
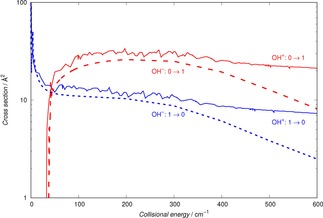
Comparing computed state changing collisional cross sections for the OH^+^/He system (present results) and for the OH^−^/He system (results from ref. [7]). See main text for further details.

The calculations which report this comparison are given by Figure 7, where the state‐changing cross sections between the lowest two rotational states of both targets are reported.

The following features of the comparison in Figure 7 are worth commenting on:

- from the energy thresholds, and up to about 300 cm^−1^ of collision energy, the two sets of cross sections are remarkably similar;
- as one goes up with the collision energy, we see that the two cross sections for the OH^−^/He case become smaller, dropping in value with similar rates as the energy increases, while the OH^+^/He cross sections remain fairly constant in value. We expect such differences to play a role in the cooling dynamics, as we shall further discuss below.

This is not an unexpected behaviour since, from the data in Figure 2, we see that the energy spacings between the lowest three rotational levels in the two molecular ions are very similar in value. Furthermore, if we compare the range of action and anisotropy of the interaction potentials, given in Figure 1 for the present cation and in our earlier reference for the OH^−^ anion,^7^ we see that the main potential coupling term comes from the long‐range part of the dipole‐polarizability contribution, which is obviously the same in both cases. Thus, at least at the low collision energies considered here, we see that the short‐range anisotropy differences between the two multipolar coefficients play a less significant role than the strong similarities between the long‐range anisotropy of the two potentials.

We therefore can say that the behaviour of the cation hydroxyl in a cold trap can be expected to show the same general behaviour as that shown earlier by the experiments with its anionic variant,^7^ a conclusion from our calculations which should bode well for the currently planned experiments on OH^+^.

## 5 State‐changing Rates at Low Temperatures

As mentioned earlier, the calculated inelastic cross sections can be now used for producing the rotationally inelastic rate constants k${}_{N\to N{}^{'}}$ (T) by numerical convolution of the computed integral partial cross sections over a Boltzmann distribution of collision energies within the experimental cold trap. In atomic units we can therefore write:

(9) $$k_{N\to N{}^{'}}\left(T\right)={\left(\frac{8}{\pi \mu k_{B}^{3}T^{3}}\right)}^{1/2}\int_{0}^{\infty }\;E\;\sigma_{j\to j{}^{'}}\left(E\right)e^{-\frac{E}{k_{B}T}}\;dE$$

where *μ* is the reduced mass of the system and *k_B_* is the Boltzmann's constant. The inelastic cross sections are those computed and discussed in the previous Section.

**Figure 8:**
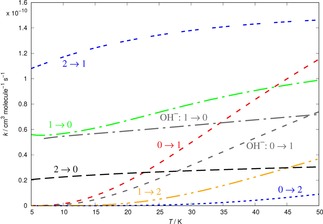
Computed state‐changing rotational rates between the lowest three rotational levels of OH^+^ in collisions with He. The two lowest rates for OH^−^/He computed earlier in ref. [7] are also shown for comparison and are given by grey dashed curves.

The computed inelastic rates for the present system, involving its lowest three rotational levels, for both the collisional “heating” and “cooling” state‐changing cases, are reported by Figure 8. We also show there a comparison with the corresponding rates for the (0→1) and (1→0) processes involving the anionic hydroxyl target.^7^

The following considerations could be made from the data in Figure 8:

- over the range of examined temperatures, the cooling rates show a fairly slow dependence on temperature, changing by at most 30 %‐40 % from 5 to 50 K;
- as hinted by the cross section behaviour discussed earlier, the (2→1) cooling rate is the largest of all cooling rates, although the three of them differ from each other by less than one order of magnitude: all rates are in the range of 10^−10^ cm^3^/sec;
- the excitation rates, on the other hand, have a more marked energy dependence when going from their threshold to the highest temperature, with the (0→1) rates increasing in value by nearly one order of magnitude from 0 to 50 K;
- in the range between 5 to 15 K, the expected optimal range of trap conditions, the whole set of rates shows a similarly slow dependence on temperature, as is also the case for the cooling rates for the OH^+^/He system.

It is also interesting to see how the present results compare with those obtained earlier for the OH^−^/He system under very similar trap conditions.^7^ In the same Figure 8 the heating and cooling rates for OH^−^ in collision with He are shown between the lowest two rotational states of the anionic target: the (1→0) rate given by a dot‐dashed gray curve, and the (0→1) rate given by short gray dashes.

We can see from the comparison that under low‐temperature trap conditions (e. g. up to about 20 K) the rates for the OH^−^ target are very close, in size and T‐dependence, to the same rates obtained in the present work for the OH^+^ molecular partners in the cold trap with He as a buffer gas. This means that state‐selective experiments for the latter are likely to evolve from their initial conditions with very similar time scales as those of the former anionic target,^7^ thus giving us some indication on the feasibility of the new experiments.

As we move to higher temperatures, however, we see that the cationic partner's rates increase with T faster than the anionic molecule, this feature indicating a swifter collisional thermalization process at higher temperatures. This particular property of the OH^+^/He system will be further analyzed in the following section.

## 6 Evaluating Relaxation Kinetics from Master Equations

The set of state‐to‐state rate coefficients discussed in the previous Section can further help us to understand the microdynamics in the trap by making use of a conventional set of rate equations (e. g. see ref.s [23, (10)]):

(10) $$dp\left(t\right)/dt=n_{He}K\left(T\right)\cdot p\left(t\right)$$

where $n_{He}$ indicates the selected density of He atoms in the trap, the vector p(t) contains the time‐evolving fractional populations of the ion partner's rotational state, p_*j*_(t), from the selected initial conditions at t=t_*initial*_, and the matrix K(T) contains the individual k_*i→j*_(T) rate coefficients at the temperature selected for the trap's conditions. Both the p(t_*initial*_) values and the collisional temperature T of the trap corresponding to the mean collisional energy between the partners (e. g. see ref.s [22, 23]), are quantitites to be specifically selected in each computational run and will be discussed in detail in the modelling examples presented below. We should note now that in the present study we shall disregard for the moment the inclusion of the state‐changing rates due to spontaneous radiative processes in the trap. These quantities are already known to be smaller than the collisionally‐controlled rates between the lower rotational levels of such systems, as already shown by us in earlier studies,^23^ and are therefore not expected to have a significant effect under the present trap conditions.

In order to cover a broad range of conditions, we have chosen an initial rotational temperature of the trap's ions to be at 400 K, so that the vector's components at t=t_*initial*_ are given by a Boltzmann distribution at that chosen temperature.

If the rate coefficients of the K(T) matrix satisfy the detailed balance between state‐changing transitions, then as t→∞ the initial Boltzmann distribution will approach that of the actual temperature of the buffer gas. These asymptotic solutions correspond to the steady‐state conditions in the trap and can be obtained by solving the corresponding homogeneous form of eq 10 given as: dp(t)/dt=0 . We solved the homogeneous equations by using the singular‐value decomposition technique (SVD),^24^ already employed by us in previous studies.^22^,^23^ The non‐homogeneous equations 10, starting from our t_*initial*_ of 400 K, were solved using the Runge‐Kutta method for different translational temperatures of the trap. Since the role of the He density is simply that of a scaling factor in the kinetics eq.s, we are presenting in these Figures only the one value which is most likely to be employed in the trap experiments.

**Figure 9:**
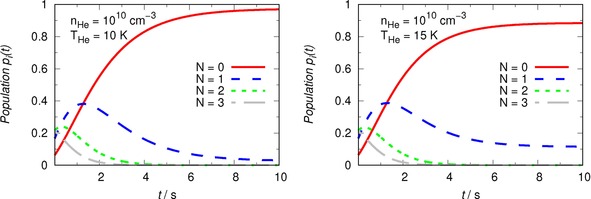
Computed time‐evolution of the rotational populations of OH^+^ molecules under different temperatures of the He buffer gas and for the experimentally expected density of the atomic partner gas. The temperature values are indicated in each panel, together with the chosen density of the buffer gas.

**Figure 10:**
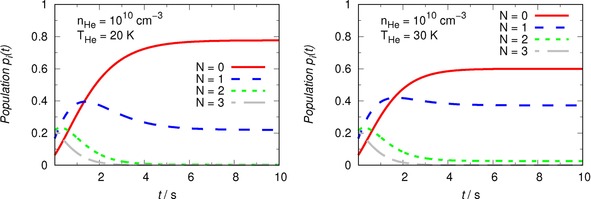
Same computed evolutionary populations as those presented in Figure 9. Here the temperature values are higher: 20 K and 30 K. See main text for a detailed discussion.

Some of the present results are shown in the sets of two panels displayed in Figures 9 and 10.

The results from the calculations described before are presented in Figures 9 for the selected value of buffer‐gas density: 10^10^ cm^−3^ . Furthermore, we have analyzed two specific low temperatures which are expected to be significant in the ion traps: 10 K (left panel) and 15 K (right panel). The following comments can be made from a perusal of these results:

- we have also run calculation tests at the lower densities of 10^9^ cm^−3^ but they are not shown in our Figures since such calculations confirmed that indeed the reduced values of collision frequencies simply cause the steady‐state population at 10 K to be reached now in nearly 100 s, while the equalization of N=0 and N=1 population around 40 % for each, takes about 15 s;
- as the density employed is the higher value expected in the ion traps (e. g.10^10^ cm^−3^) and shown in the two panels of the Figure, we see that the steady state populations are reached in about 10 s, nearly one order of magnitude faster, as expected by simple scaling criteria;
- on the other hand, as the temperature increases from 10 K to 15 K, we see that the N=1 population remains still substantial after reaching the steady‐state conditions.

The additional data in the two panels of Figure 10 emphasize the marked effects from introducing in the trap increasingly “hotter” He gas while considering the same buffer gas density explored in Figure 9.

Thus, we see that 10^10^ cm^−3^ is definitely an effective He density in the trap, since at all temperatures the rotational populations reach steady‐state conditions within about 10 s. On the other hand, as the temperature increases, we see that the populations of the N=1 rotational state becomes increasingly more important, thereby reducing the relative population values of the ground rotational state of the cationic target.

We further see from the Figures that, as the trapped molecules thermalize to increasingly larger temperatures of the buffer gas, the final populations of the N>0 rotational states naturally increase and leave fewer molecules into the N=0 state of OH^+^.

**Figure 11:**
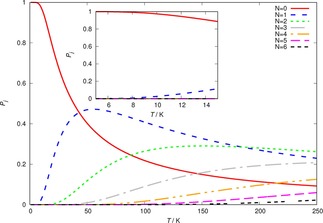
Steady‐state solutions of the kinetic eq. 10 as a function of trap temperature from the He buffer gas. The inset shows an enlargement of the data within the low temperature region.

To verify the numerical accuracy of the present solutions of the kinetics eq.s, we report in Figure 11 the steady‐state populations of some of the relevant rotational states of the molecular cation as we increase the temperature of the trap.

It is interesting to note that the population changes with temperature produce, at each T value, results which essentially coincide with the Boltzmann's distribution at that temperature. This result agrees with what we had already verified in our earlier studies.^22^,^23^ In those papers we had also made use of another useful indicator for assessing the efficiency of the time‐dependence of the process under study. By defining a characteristic time constant, τ , which is given as:

(11) $$\left\langle E_{rot}\right\rangle \left(\tau \right)-\left\langle E_{rot}\right\rangle \left(t=\infty \right)=\frac{1}{e}\left(\left\langle (E_{rot}\right\rangle \left(t=0\right)-\left\langle E_{rot}\right\rangle \left(t=\infty \right)\right)$$

here $\left\langle E_{rot}\right\rangle$ represents the level‐averaged rotational energy of the molecules in the trap after a characterizing time interval defined by the index τ of above. The latter quantity also depends on collision frequencies and is therefore, as it is to be expected, inversely proportional to the n_*He*_ in the trap. It also turn out to be only a slow function of the trap temperature in the region of T values of interest for the present modeling.

**Figure 12:**
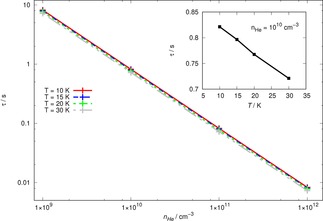
Computed characteristic relaxation times of OH^+^ molecules in the trap, as a function of the density of the He buffer gas. The inset on the upper left of the Figure shows an enlargement around the density value of 10^10^ cm^−3^.

We report in Figure 12 the dependence of the computed τ values on the variation of the density for the buffer gas and for four different values of trap temperatures

It is interesting to note that the selection of the optimal density for the uploaded buffer gas is clearly a very important step for the present system. When we make n_*He*_ vary by four orders of magnitude in the calculations, we see that the characteristic relaxation times (at four different temperatures) vary by three orders of magnitude. From the enclosed inset we also see that the variation with the temperature, shown around one specific n_*He*_ value, is rather slow. In any event, when selecting the buffer gas density around 10^10^ cm^−3^, not a very taxing demand on the experiments, the values of τ are less than a second which indicates a rather efficient relaxation kinetics for the present system.

One should remember, in fact, that the value of τ measures a relaxation process that indicates an averaging over the available states at the chosen temperature, i. e. the time taken by collisional processes to thermalize the present trapped ions to the selected temperature for the uploaded buffer gas. Thus, our calculations indicate that in less than one second we could bring the OH^+^ molecules to be distributed among their rotational states that are linked to a Boltzmann's distribution for the temperature reached by the uploaded buffer gas.

## 7 Present Conclusions

In this work we have analyzed in some detail the collisional dynamics of rotational state‐changing processes involving the lower rotational states of a trapped molecular ion, the OH^+^(${}^{3}\Sigma^{-}$ ) molecule, by uploading into the trap He atoms as the buffer gas.

The analysis has covered several steps of the *ab initio* treatment of the quantum inelastic dynamics: we have first selected an accurate electronic potential energy surface by comparing our earlier quantum chemical study^14^ which had been carried out in our group, with a more recent calculation of the same potential using an ab initio evaluation^13^ that turned out to be very similar to the one from our earlier study.

We have then employed the chosen PES to calculate the inelastic, state‐to‐state partial integral cross sections involving the lowest four rotational state of the OH^+^(${}^{3}\Sigma^{-}$ ) molecule. Our calculations confirmed the earlier findings and further show that it is possible to generate very accurately inelastic, state‐averaged, cross sections for which the target molecule is viewed as a pseudo‐singlet, closed‐shell target molecule. Thus the spin‐rotation structures can be initially disregarded and the states of the target being labelled solely by the rotational quantum number N.

From the computed cross sections we further obtained the inelastic, state‐to‐state collisional rates over a broad range of trap temperatures. We discovered that our present inelastic rates are very similar in size to the same rates for the OH^−^/He system which has been extensively analyzed in our group.^7^ In fact, our present calculations show that both systems present very similar temperature dependence of their rates when the trap temperature is taken to be between 5 and 20 K, not a very strong demand on experimental methods.^7^

We finally have employed the computed rates to solve the corresponding kinetics eq.s in order to obtain the time evolution of the rotational states of the trapped OH^+^ molecules by uploading into the trap increasingly colder He atoms, further confirming the expected scaling role of having variable density values associated with them.

The characteristic relaxation times for a state‐averaged time evolution process indicate that the OH^+^ trapped molecules should effectively thermalize to a selected buffer gas temperature of the traps (ideally between 5 and 15 K) within less that one second, provided the density of the buffer gas is kept around 10^10^ cm^−3^, which in not a very strong experimental requirement.^22^

## Conflict of interest

The authors declare no conflict of interest.

## Supporting information

As a service to our authors and readers, this journal provides supporting information supplied by the authors. Such materials are peer reviewed and may be re‐organized for online delivery, but are not copy‐edited or typeset. Technical support issues arising from supporting information (other than missing files) should be addressed to the authors.

Supplementary
