# Supplementary material for: Modeling Quantum Kinetics in Ion Traps: State‐changing Collisions for OH+(3Σ- ) Ions with He as a Buffer Gas
Source: Chemphyschem. 2018 Jun 21;19(15):1866–75. doi: 10.1002/cphc.201800119 (PMC6099509; doi:10.1002/cphc.201800119)
Supplement: Supplementary file 1 — Supplementary [file CPHC-19-1866-s001.pdf]

Author Contributions

F.G. Writing – original draft:Lead
R.W. Writing – review & editing:Supporting
